# Identification of the Merkel Cell Polyomavirus Large Tumor Antigen Ubiquitin Conjugation Residue

**DOI:** 10.3390/ijms22137169

**Published:** 2021-07-02

**Authors:** Luz E. Ortiz, Alexander M. Pham, Hyun Jin Kwun

**Affiliations:** 1 Department of Microbiology and Immunology, Pennsylvania State University College of Medicine, Hershey, PA 17033, USA; lguevara@pennstatehealth.psu.edu (L.E.O.); amp6528@psu.edu (A.M.P.); 2 Penn State Cancer Institute, Hershey, PA 17033, USA

**Keywords:** Merkel cell polyomavirus, Merkel cell carcinoma, large tumor antigen, Ubiquitination, ATPase activity, Polyomavirus replication

## Abstract

Merkel cell polyomavirus (MCPyV) large tumor (LT) antigen is a DNA binding protein essential for viral gene transcription and genome replication. MCPyV LT interacts with multiple E3 ligases in a phosphorylation-dependent manner, limiting its own viral replication by enhancing LT protein degradation, which is a unique mechanism for MCPyV latency. Thus, identifying LT ubiquitination sites is an important step toward understanding the biological role of these virus-host interactions that can potentially result in viral oncogenesis. The ubiquitin (Ub) attachment sites in LT were predicted by using Rapid UBIquitination (RUBI), a sequence-based ubiquitination web server. Using an immunoprecipitation approach, the lysine (Lys, K) 585 residue in LT is identified as the ubiquitin conjugation site. Lysine 585 is deleted from tumor-derived truncated LTs (tLTs), resulting in stable expression of tLTs present in cancers. Substitution of lysine 585 to arginine (Arg, R) increased LT protein stability, but impaired MCPyV origin replication, due to a loss of ATP hydrolysis activity. These findings uncover a never-before-identified ubiquitination site of LT and its importance not only in the regulation of protein turnover, but also in MCPyV genome replication.

## 1. Introduction

Ubiquitination is the reversible process by which a protein is post-translationally modified by the covalent conjugation of one or more 8.5-kilodalton (kDa) ubiquitin (Ub) molecules to specific lysine (Lys, K) residues on substrate proteins. The common outcome of these proteins is their later degradation by the 26S proteasome complex [1,2]. Attachment of Ub moieties to target proteins takes place in a series of enzymatic reactions that can be summarized in three steps, where Ub is activated by E1 (Ub-activating protein), transferred to the E2 (Ub-conjugating protein), and then attached to the target substrate by E3 (Ub-ligase protein) [3]. Recent studies have demonstrated that the ubiquitin-proteasome pathway provides a layer of antiviral property by having cellular E3 ligases targeting essential proteins for viral replication, suppressing their stability and function via proteasomal-enhanced degradation [4]. This can be seen in Influenza A virus (IAV) [5], Hepatitis B virus (HBV) [6,7], Hepatitis C virus (HCV) [8], Japanese encephalitis virus (JEV) [9], and Human Papillomavirus (HPV) [10]. It is clear that the ubiquitin-proteasome pathway impacts multiple steps in the virus life cycle. Systematic dissection of the ubiquitination proteome in virus infection is expanding; however, it is a challenging research area, since ubiquitination not only plays a role in protein degradation, but also in many other cellular functions that are complexly regulated. Therefore, identifying the specific substrates of each E3 ligase and determining their ubiquitination sites are important for understanding various biological events, including host-virus interactions.

**Figure 1:**
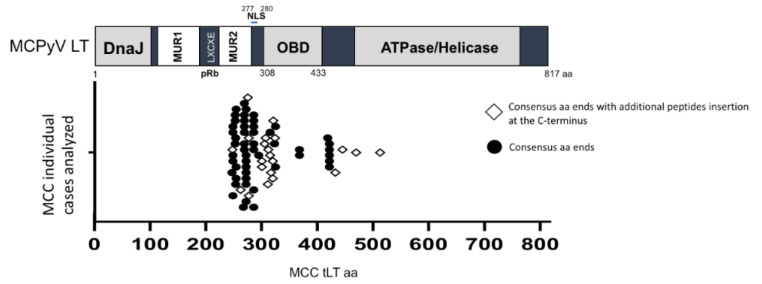
MCC-derived MCPyV strains have mutations resulting in the truncation of LT C-terminus ATPase/helicase domain. MCPyV LT antigen contains multiple domains, including the DNA J domain, disordered MCPyV unique region (MUR) divided into two fragments by the LXCXE sequence, a pRb-binding motif [13,14], the origin-binding domain (OBD), and the ATPase/helicase domain. NLS, nuclear localization signal [20]. Sequences from 72 MCC tLTs deposited in the GenBank were analyzed to distribute tLT protein sizes (sequence length) (Table S1). The graph depicts the distribution of all 72 tLT sizes and the distinction between those with additional nonconsensus oligopeptides at their C-termini.

Merkel cell polyomavirus (MCPyV), an oncogenic double-stranded DNA virus, is the causative agent of Merkel cell carcinoma (MCC), a rare but lethal skin cancer with a high propensity to metastasize, disproportionally affecting immunosuppressed and elderly individuals [11]. Monoclonal integration of the MCPyV genome into the DNA of tumor cells is found in about 80% of MCC cases [12]. One of the MCPyV early gene transcripts, the large tumor (LT) antigen, is an 817-amino acid (aa) protein that contains functional regions, including the DNA J domain, the disordered MCPyV unique region (MUR) divided into two fragments by the LXCXE sequence, the retinoblastoma tumor suppressor (Rb)-binding motif [13,14], the origin-binding domain (OBD) and the ATPase/helicase domain (Figure 1). MCPyV LT is known to interact with multiple Skp-Cullin-F box (SCF) E3 ubiquitin ligases [4] and ubiquitin-specific-processing protease 7 (USP7) [15] both involved in the ubiquitin-proteasome pathway. MCPyV full-length LT is required for viral replication, since it recognizes its viral origin to initiate replication of the MCPyV genome [16,17]. Interestingly, MCPyV found in cancer presents truncated LTs (tLTs), due to premature nonsense mutations. These tumor-derived tLTs are characterized by the truncation of the ATPase/helicase domain in the C-terminus [14], which results in the impairment of the virus to replicate its own genome and render the virus untransmissible.

To investigate the role of ubiquitination and its impact on MCPyV LT function, we used a computational prediction approach and identified the lysine 585 residue, located within the ATPase/helicase domain, as the site responsible for LT ubiquitination. The question that followed was whether K585 ubiquitination affected viral genome replication and LT protein stability. Sequence analysis of different MCC-derived tLTs demonstrated the loss of K585 in all analyzed tLTs. Mutation of K585 to arginine (Arg, R) significantly inhibited LT protein degradation, and resulted in the loss of virus replication function. A structural prediction of the ATPase domain in LT showed that K585 is homologous to K418, one of the ATP “locker” residues, in Simian virus 40 (SV40) LT located within the ATP binding pocket [18,19]. MCPyV LT K585 also played an important role in ATP hydrolysis required for helicase activity and viral replication. Our findings show that the ubiquitin-proteasome pathway plays a key role during MCPyV infection and replication, and characterize for the first time a ubiquitin-attachment site in MCPyV LT.

## 2. Results

### 2.1. Protein Stability of MCC-Derived tLTs

MCPyV LT protein stability seems to be a key determinant in regulating host cell growth [13]. To date, the C-terminus of all MCC-derived tLTs are known to be deleted by nonsense mutations occurring during oncogenic progression. However, it is not known whether the protein stability of tLTs is affected by the deletion of the C-terminal ATPase/helicase domain. MCPyV LT truncating mutations found in MCCs result in the expression of different length tLT proteins. We first compiled and analyzed the tLT amino acid sequences of 72 MCC cases deposited in the GenBank database to gain knowledge of the most frequent tLT sizes (Table S1). The spectrum of tLT mutations is quite broad, but they mainly occur between amino acids 254–431. In 21 MCC cases analyzed, tLTs have acquired additional nonconsensus oligopeptide sequences at their C-termini (Figure 1).

**Figure 2:**
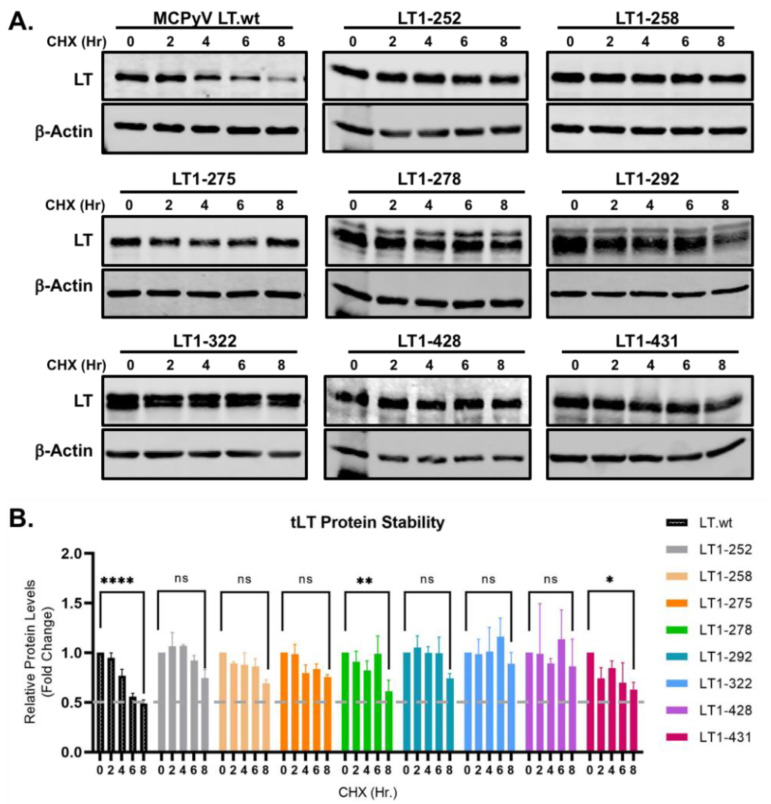
MCC-derived tLTs are stably expressed. (**A**) Eight tLT constructs with consensus amino acid ends, generated by nonsense mutations were tested for their stability. LT protein turnover was assessed by a cycloheximide (CHX) chase assay and measured using quantitative immunoblot analysis. HEK293 cells transfected with either LT.wt or tLT constructs (0.5 μg) were treated with CHX (0.2 mg/mL) at 24 h after transfection and harvested at each time point indicated. (**B**) Protein expression was quantified using a laser-scanning Odyssey CLX (LI-COR) infrared (IR) imaging system. Mean values, error bars representing the standard error of the mean (SEM), and *p*-values between time 0 and 8 h were calculated using GraphPad Prism software (**** *p* ≤ 0.0001, ** *p* = 0.0078, * *p* = 0.0233, ns = not significant). Data were analyzed using three biological replicates per experiment, *n* = 3.

To determine the protein turnover of tLTs, we generated eight tumor-derived tLT constructs containing consensus amino acid ends generated by nonsense mutations (1–252, 1–258, 1–275, 1–278, 1–292, 1–322, 1–428, 1–431 aa) and transfected them into HEK293 cells. Their protein stability was tested using a cyclohexamide (CHX) chase assay. Samples were harvested at multiple time points over the course of 8 hours (hr, h), and LT protein was detected using 2B4 antibody (Figure 2A). Protein expression was quantified, normalized to β-actin, and compared to wild-type LT (LT.wt) (Figure 2B). All tLTs have significantly longer half-lives (t_1/2_ ≥ 8 h) than LT.wt (t_1/2_ = ~6 h), indicating that the C-terminal domain modulates the susceptibility of LT to degradation.

### 2.2. Lysine 585 in LT Is Required for LT Ubiquitination

**Figure 3:**
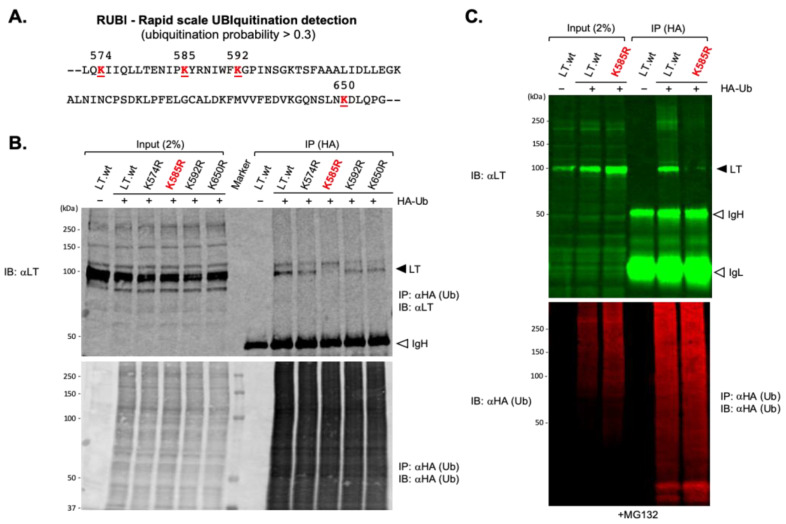
The ubiquitin conjugation lysine residue of MCPyV LT. (**A**) Potential LT ubiquitination lysine residues from 431 to 817aa were predicted using Rapid UBIquitination (RUBI), a sequence-based ubiquitination predictor. Four lysine residues in LT with ubiquitination probability over 0.3 are shown. Each lysine residue was substituted with arginine. (**B**) K585 is the ubiquitin conjugation site of MCPyV LT. HEK293 cells expressing either MCPyV LT protein or lysine mutants together with HA-Ub, as indicated, were immunoprecipitated with HA-conjugated agarose and immunoblotted for LT. Samples were loaded onto 8% SDS polyacrylamide gel. K585R mutant diminished its ability to interact with Ub. IgH = immunoglobulin heavy chain. (**C**) HEK 293 cells co-transfected with either Lt.wt or K585R mutant and HA-Ub were treated with MG132 (10 μM). HA-Ub proteins were immunoprecipitated with HA-conjugated agarose. Precipitated proteins were resolved using 4–20% Criterion TGX precast gradient gels (Bio-Rad) and analyzed by quantitative immunoblotting. IgH = immunoglobulin heavy chain, IgL = immunoglobulin light chain.

The N-terminal MUR domain of MCPyV LT interacts with multiple SCF E3 ubiquitin ligase complexes that ubiquitinate and trigger proteasomal degradation of LT [4,13]. To date, no ubiquitin attachment site of LT has been identified. Although tLTs contain the MUR E3 ligase binding domain, they maintained their increased protein stability, indicating that the C-terminus in LT may play a role in ubiquitin conjugation. To analyze the involvement of the C-terminal domain in LT ubiquitination, we used Rapid UBIquitination (RUBI), a sequence-based ubiquitination predictor to identify potential LT ubiquitination sites at the C-terminus (431 to 817 aa). Four lysine residues (K574, K585, K592, and K650) with ubiquitination probability over 0.3 were selected for further tests (Figure 3A). Then each lysine residue was substituted for arginine.

To assess the potential interaction of these predicted lysine residues with Ub, either LT.wt or each of the four lysine mutants were co-expressed along with HA-tagged ubiquitin (HA-Ub) in HEK293 cells and immunoprecipitated from cell lysates using an affinity resin specific to the HA tag. One major band migrating around 100 kDa, consistent with LT protein with the addition of a single 8.5-kDa HA-ubiquitin molecule, was detected on immunoblots probed with anti-LT antibody (2B4) (Figure 3B). These bands could not be detected in lysates from cells expressing LT.wt alone, indicating that these bindings are specific for ubiquitinated LTs. The LT-Ub conjugate was significantly reduced when K585 in LT was substituted with R585 (Figure 3B,C), indicating that K585 is the residue where ubiquitin attachment takes place.

### 2.3. Lysine 585 Regulates LT Stability

**Figure 4:**
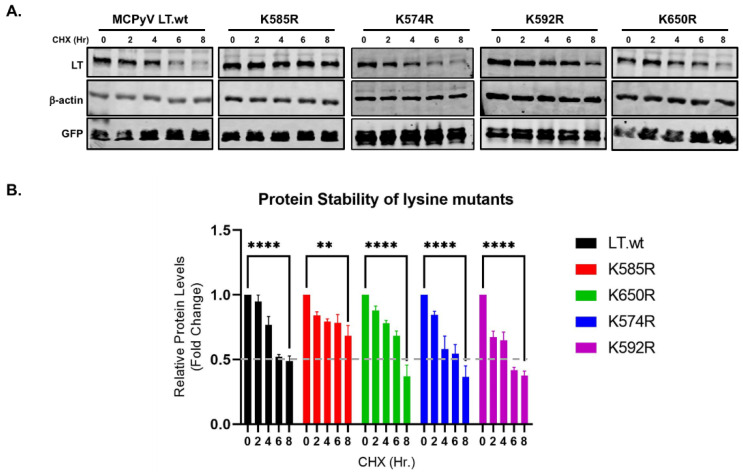
LT K585 destabilizes LT. (**A**) Mutation of K585 to R significantly increased LT stability. HEK293 cells were transfected with either LT.wt or each of the LT mutant constructs and treated with CHX (0.2 mg/mL) to inhibit new protein synthesis. Cells were harvested at each time point indicated. (**B**) Protein expression was quantified using the laser-scanning Odyssey CLX (LI-COR) infrared (IR) imaging system. Mean values, error bars representing the standard error of the mean (SEM), and *p*-values between time 0 and 8 h were calculated using GraphPad Prism software (**** *p* ≤ 0.0001, ** *p* = 0.0022). Data were analyzed using three biological replicates per experiment, *n* = 3.

Our previous data concluded that the loss of the ubiquitin conjugation site at the C-terminus in LT rendered the LT protein resistant to degradation, as shown in Figure 2. In addition, since the K585R mutation prevents the attachment of Ub to LT (Figure 3), it was only logical to think this mutation would also prevent degradation of the LT protein. To verify if the K585R mutant affected LT degradation, we expressed either LT.wt or each of the four lysine mutants in HEK293 cells. LT protein stability was analyzed using a cycloheximide (CHX) chase assay and quantitative immunoblot analysis. As shown in Figure 4, mutation of K585 to R significantly reduced LT protein turnover (t_1/2_ > 8 h) in comparison to wild-type and the other three lysine mutants, as expected.

### 2.4. Lysine 585 Ubiquitination Regulates LT-Mediated MCPyV Replication

**Figure 5:**
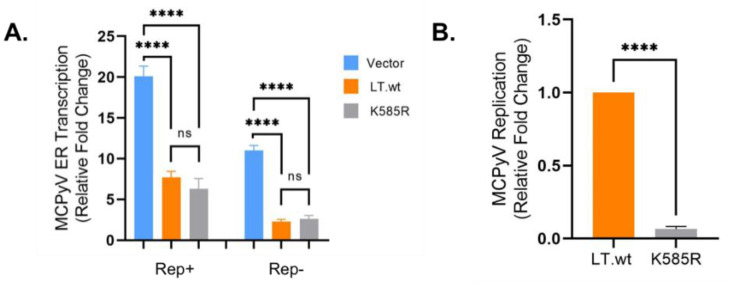
LT K585 is required for MCPyV replication. (**A**) LT K585R mutation maintains origin-binding ability. Early gene (ER) transcription activity was measured by luciferase activity. The reporters (Rep+ and Rep-, 0.5 μg) [4] were respectively transfected with either LT.wt or LT K585R mutant (0.5 μg). LT K585R similarly downregulated MCPyV ER transcription compared to LT.wt. Mean values, error bars representing the standard error of the mean (SEM), and *p*-values were calculated using GraphPad Prism software (**** *p* ≤ 0.0001, ns = not significant). Data were analyzed using three biological replicates, *n* = 3. (**B**) K585 is required for MCPyV viral replication. MCPyV origin replication was determined from samples tested in Figure 5A (Rep+). Episomal DNAs were isolated and digested with DpnI. Replicated origin DNA was measured by qPCR. Mean values, error bars representing the standard error of the mean (SEM), and *p*-value were calculated using GraphPad Prism software (**** *p* ≤ 0.0001). Data were analyzed using three biological replicates per experiment, *n* = 3.

There are diverse functions associated with protein ubiquitination [2]. Being that K585 is located within the DNA/helicase domain, we sought to determine if LT ubiquitination affected LT ability to regulate transcription/DNA-binding and/or MCPyV origin replication. Dual bidirectional reporter constructs (Rep+ and Rep-) [4] were used to measure MCPyV early gene (ER) transcription and viral replication. A reporter was co-transfected with either LT.wt or K585R mutant into HEK293 cells. Firefly luciferase activity was measured to examine the effect on LT-DNA binding capacity and ER transcription. LT.wt repressed MCPyV ER transcription, as previously reported [4,13]. K585R mutant also downregulated MCPyV ER transcription to similar levels, as well as LT.wt, when the replication-competent reporter (Rep+) was used. Comparable results were observed using the replication-deficient reporter (Rep-) [4,13], indicating that LT ubiquitination does not affect the DNA-binding ability of LT (Figure 5A). However, K585R significantly reduced the LT capacity to replicate the MCPyV origin (Figure 5B), as it retained its ability to bind the origin DNA.

### 2.5. Lysine 585 Is an ATP-Binding Site That Regulates ATP Hydrolysis for Viral Replication

**Figure 6:**
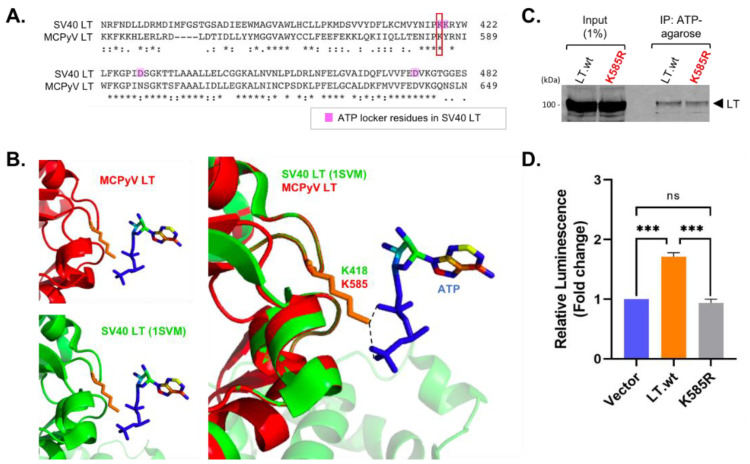
MCPyV K585 similarly localizes as one of ATP locker residues, K418, in SV40 LT. (**A**) SV40 LT ATPase sequence (363–482 aa) was compared with MCPyV LT (534-649 aa) (Clustal Omega). MCPyV LT K585 residue corresponds to the K418 in SV40 LT. (**B**) MCPyV LT ATPase domain (440–792 aa prediction, 100 confidence, 48% identity) structure (red) was predicted using a Phyre2 web server [22] based on SV40 LT structure (1SVM) (green) [18,19]. MCPyV LT K585 similarly localizes as K418 in SV40 LT for ATP binding (orange). (**C**) K585R mutation does not change the effect on ATP binding, due to a positive electrostatic potential. MCPyV LT.wt and K585R mutant were overexpressed in HEK293 cells and pulled down using ATP-agarose resin. (**D**) LT ATP hydrolysis activity was measured using the ADP-Glo assay. The K585R point mutation abolishes LT ATP hydrolysis activity. Mean values, error bars representing the standard error of the mean (SEM), and *p*-values were calculated using GraphPad Prism software (*** *p* = 0.0002 (V:LT), *** *p* = 0.0001 (LT:K585R), ns = not significant).

SV40 LT has been extensively studied and used as the prototype for eukaryotic DNA replication, due to the well-defined structural modeling of its origin of replication. SV40 LT DNA binding and ATPase/helicase domain structures have been well characterized. To compare structure and sequence variations between SV40 LT and MCPyV LT ATPase domains, the MCPyV LT ATPase domain (440–792 aa) structure was modeled by Phyre 2 web portal (100 confidence, 48% i.d. with 1SVM [19]). The ClustalW alignment of the amino acid sequences between SV40 LT and MCPyV LT ATPase domains showed that K585 in MCPyV LT is homologous to K418 in SV40 LT. Previous structure studies of SV40 LT ATPase domain identified the K418 in SV40 LT as one of the ATP “locker” residues required to ensure the proper ATP binding and its subsequent hydrolyzation [18] (Figure 6A,B). To determine whether K585 is required for the ATP binding and ATP hydrolysis necessary for the helicase activity, we expressed either LT.wt or K585R mutant in HEK293 cells and then pulled down using ATP-agarose beads (Figure 6C). Both LT.wt and K585R mutant efficiently bound to ATP, possibly due to the positive electrostatic potential present in both K and R residues which attract the negatively charged phosphate group of ATP [21]. Next, we performed ATPase activity assays to examine the function of K585 in viral replication. Mutant K585R abolished the LT ATPase hydrolysis activity to levels comparable to empty vector. This was measured by quantifying the luminescence signal of ADP levels converted from ATP, suggesting that ubiquitin conjugation at K585 in MCPyV LT is critical for MCPyV replication (Figure 6D).

## 3. Discussion

For decades, studies of polyomavirus large T focused mainly on their oncogenic functions, due to the discovery of the SV40 LT antigen role in human cell transformation [23]. Although viruses commonly exploit cellular ubiquitination machinery to enhance cell proliferation and to antagonize antiviral responses, knowledge of the interplay between pathogens and the cellular ubiquitin system remains limited. SV40 LT protein is highly stable (t_1/2_ ≥ 36 h) compared to MCPyV LT(t_1/2_ = ~4–6 h), and acetylation is known to be a predominant modification in SV40 LT rather than ubiquitination [24]. Indeed, no ubiquitin conjugation site has ever been identified before in any other polyomavirus LT studies. Our results identify, for the first time, a ubiquitin conjugation site in polyomavirus LT protein and characterize potential functions of ubiquitination in virus replication.

While wild-type MCPyV LT represses cell growth, all MCC tumor-derived LT antigens have shown to be truncated at the C-terminus, which promotes cell growth. These truncations impair their own DNA binding ability, ATPase/helicase activity, and DNA damage response (DDR) which have a negative impact on cell proliferation [25]. Here we show that tumor-derived tLTs do not possess the K585 residue, enabling tLTs to stably target tumor suppressors, such as pRb, for oncogenic progression [14]. Interestingly, some of the tLTs carry additional oligopeptide sequences that are generated by a combination of missense/nonsense mutations at the C-terminal. It is possible that these additional nonconsensus peptide sequences could modulate their tLT stability or result in other modifications that can affect the clinical outcome. Nonetheless, the majority of tLTs have lost most of their DNA-binding/oligomerization capacity, and 44% of tLTs analyzed here (32 out of 72 cases) are missing the nuclear localization signal (NLS) [20]. In these tumor cases, loss of the NLS may cause different geometric characteristics of protein-protein interactions compared to the wild-type LT in addition to its changes in stability and structure.

In this study we identified and characterized the ubiquitin conjugation lysine residue in MCPyV LT. It is still not clear if LT is mono -or polyubiquitinated. Either way, both mono- and multi-ubiquitinated proteins are commonly processed by proteasomes [26]. Although ubiquitination is mainly considered to be important for LT function and stability, other regulatory mechanisms or modifications may exist. To our surprise, the LT ubiquitination site functionally overlaps with the ATP binding domain. This suggests that LT ubiquitination may interfere with ATP binding required for virus replication. These observations contribute to our previous interaction studies of LT with multiple E3 ligases [4,13,27], since they not only promote LT turnover, but also may inhibit MCPyV replication by enhancing LT ubiquitination, which needs further investigation.

Studies of the interactions between E3 ligases and substrate viral proteins have been emerging to understand the role of the protein ubiquitination regulatory network in pathogenesis. Our result specifically defines a direct role of ubiquitination in virus replication that has not been determined before.

## 4. Materials and Methods

### 4.1. Cell Culture and Transfection

HEK293 cells were cultured in DMEM with 10% premium grade fetal bovine serum (FBS) (Avantor Seradigm, Radnor, PA, USA). Transfections with expression vectors were performed using jetOPTIMUS (Polyplus Transfection, New York, NY, USA) according to the manufacturer’s instructions.

### 4.2. Plasmids

Codon-optimized, commercially synthesized MCPyV LT antigen sequences were cloned into pcDNA6/V5/His vector (Invitrogen, Waltham, MA, USA) with a modified multiple cloning site (MCS) (Addgene #40200, Watertown, MA, USA) [27]. All LT mutations were generated by overlapping PCR mutagenesis. MCPyV LT K574R, K585R, K592R, K650R mutants were generated using primer pairs in Table S2. Truncated LT sequences (codon-optimized) were generated by PCR using primer pairs (Table S2) and inserted into AfeI and BamHI sites of pLVX-puro empty vector. pRK5-HA-Ubiquitin-WT plasmid was obtained from Addgene (#17608) [28].

### 4.3. Quantitative Immunoblotting, Immunoprecipitation, and Antibodies

LT protein turnover was measured by a cycloheximide (CHX) chase assay using quantitative immunoblot analysis. LT plasmids were co-transfected with an eGFP plasmid [29] to normalize transfection efficiency, and all experiments were performed in triplicate. Cells were lysed in IP buffer (50 mM Tris-HCl (pH 7.4), 150 mM NaCl, 1% TritonX-100, 1 mM PMSF, 1 mM benzamidine). Whole cell lysates without preclearing were used for direct immunoblotting. For detecting ubiquitinated LT protein, HEK293 cells expressing LT and pRK5-HA-Ubiquitin-WT were lysed in IP buffer, and the lysates were incubated with HA-conjugated agarose (Pierce Biotechnology, Rockford, IL, USA) overnight at 4 °C. Precipitated proteins were resolved by SDS–polyacrylamide gel electrophoresis (PAGE) using 8% or 4–20% Criterion TGX precast gradient polyacrylamide gels (Bio-Rad Laboratories, Hercules, CA, USA). Protein interaction was analyzed by quantitative immunoblotting using the infrared (IR) fluorescence-based detection, a western blot assay specifically designed to establish the dynamic linear range of detection. These quantitative comparisons should not be made in the saturated range of protein loading using nonproportional or nonlinear models, such as enzymatic detection methods. Primary antibodies were incubated overnight at 4 °C, followed by 1 h secondary antibody incubation at room temperature (RT). All signals were detected using quantitative IR secondary antibodies (IRDye 800CW goat anti-mouse, 800CW goat anti-rabbit, 680LT goat anti-rabbit IgG, 680LT goat anti-mouse IgG) (LI-COR, Lincoln, NE, USA). Signal intensities were analyzed using a laser-scanning imaging system, Odyssey CLX (LI-COR). A one-way analysis of variance (ANOVA) and two-tailed unpaired t test were used to determine statistical significance using GraphPad Prism software. Anti-MCPyV large T-antigen (2B4, Santa Cruz Biotechnology, Dallas, TX, USA), GFP (D5.1, Cell Signaling Technology, Danvers, MA, USA), HA-Tag (C29F4, Cell Signaling Technology), β-Actin (13E5, Cell Signaling Technology) antibodies were used for this study.

### 4.4. MCPyV ER Transcription Analysis

Dual bidirectional reporter constructs were used to measure MCPyV gene transcription (Rep+ and Rep- reporters) [4]. The activity of the Firefly (early gene reporter) luciferase was determined using the luciferase reporter assay (Promega Corporation, Madison, WI, USA) according to the manufacturer’s protocol. A plasmid expressing eGFP [29] was used as an internal transfection efficiency control. Luminescence and eGFP fluorescence were analyzed using a Synergy 2 fluorescence reader (Biotek, Winooski, VT, USA). The levels of the experimental reporter activity (luminescence) were normalized to the eGFP fluorescence intensity. Differences between means (*p*-value) were analyzed using a t-test with GraphPad Prism software.

### 4.5. MCPyV Origin Replication Assay

HEK293 cells were transfected with an LT expression vector (LT.wt) and Rep+ (MCPyV ER transcription reporter) using jetOPTIMUS (Polyplus Transfection) in 12-well plates. Episomal DNA was collected by salt-precipitation at 48 h post-transfection. 1 μg of DNA was digested with DpnI, then 50 ng of digested DNA was subjected to qPCR. qPCR was carried out with PowerUp^TM^ SYBR Green Master Mix (Applied Biosystems, Foster City, CA, USA) using a StepOnePlus system (Applied Biosystems) according to the manufacturer’s protocol. A primer pair (5′-CAACTTGGCTGCCTAGGTG-3′, 5′-CTTGTCTATATGCAGAAGGAGTTTGCAG-3′) was used for MCPyV origin detection.

### 4.6. ATP Binding and ATPase Activity Assay

HEK293 cells transfected with LT constructs were treated for 24 h with the glucose-deprived medium. LT proteins (LT.wt and K585R) expressed in HEK293 cells were incubated overnight at 4 °C with ATP agarose (Sigma-Aldrich, St. Louis, MO, USA) in IP buffer [4]. The agarose beads were washed with IP buffer and high-salt buffer (0.5 M LiCl in 50 mM Tris, pH 7.6) and then resuspended in 2×SDS loading buffer for immunoblots. For ATPase activity of LT constructs, LT proteins (LT.wt and K585R) expressed in HEK293 cells were immunoprecipitated using 2 μg of 2B4 antibody with Protein A/G Plus resin (Santa Cruz Biotechnology). Approximately 40 μg of elutes of each sample were mixed with 1 ng of dsDNA containing MCPyV origin (nt 5154–195, GenBank: EU375804.1) in a buffer containing 20 mM Tris-Cl pH 7.5, 50 mM NaCl, 0.1 mM EDTA, 0.1 mg/mL BSA and 1 mM DTT. The mixture was incubated for 60 minutes (min) at RT. Then, final 100 μM ATP (New England Biolabs, Ipswich, MA, USA) was added to the mixture and incubated for 30 min. After adding 10 mM MgCl_2,_ samples were incubated at 37 °C for 45 min. The reaction mixture was used to measure the conversion of ATP to ADP using the ADP-Glo assay kit (Promega) according to the manufacturer’s protocol. Statistical significance was determined using the one-way ANOVA test using the GraphPad Prism software.

### 4.7. Ubiquitination Site Prediction and Structural Analysis

Rapid UBIquitination (RUBI) [30], a sequence-based ubiquitination predictor designed for rapid application on a genome scale was used to predict MCPyV LT ubiquitination sites. Lysines with ubiquitination probability over 0.3 were selected for further stability testing. MCPyV LT ATPase domain (440–792 amino acids) structure was modeled by Phyre 2 web portal (100 confidence, 48% i.d.) [22]. Protein data bank (PDB) structure 1SVM was used and compared [19] for ATP binding prediction (Figure 6). Ribbon representations, as well as structural alterations, were achieved with the PyMOL program.

## 5. Conclusions

Our study identified and characterized for the first time a ubiquitin conjugation site of a polyomavirus LT, demonstrating a functional role of MCPyV LT ubiquitination in the regulation of LT protein turnover and virus replication, which could be a key feature leading to the development of MCC.

## Data Availability

The data presented in this study are available in figures and supplementary file.

## Supplementary Materials

The following are available online at https://www.mdpi.com/article/10.3390/ijms22137169/s1, Table S1: LT mutations in MCC; Table S2: Primers for LT constructs.
